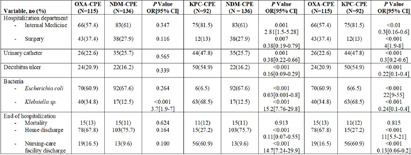# A Decade of Change: Shifting Trends in Carbapenemase-Producing Enterobacterales Among Hospitalized Patients

**DOI:** 10.1017/ash.2025.415

**Published:** 2025-09-24

**Authors:** Lisa Saidel-Odes, Seada Eskira, Jan Feldman, Alexander Goshansky, Orli Sagi, Shani Troib, Borer Abraham

**Affiliations:** 1 Soroka University Medical Center; 2 soroka; 3 soroka medical centre; 4 Soroka University Medical Center; 5 SOROKA MEDICAL CENTER

## Abstract

**Background:** Carbapenemase-producing Enterobacterales (CPE) poses a major infection control challenge in healthcare settings. Over the past decade, Klebsiella pneumonia carbapenemase (KPC)-CPE colonization at our hospital declined to under 10% of all CPE rectal screens, while New Delhi metallo-beta lactamase (NDM)-CPE and oxacillinase (OXA)-CPE colonization rates have tripled, Figure 1. **Methods:** A comparative historical study was conducted on adult patients colonized with OXA-CPE (2017-2023), NDM-CPE (2017-2023), or KPC-CPE (2017-2018). Patients were retrospectively identified through the microbiology laboratory, their files reviewed for demographics, clinical characteristics, and outcomes. **Results:** The study included all 341 patients who underwent a screening rectal swab for CPE on admission or during contact tracing: 115 tested positive for OXA-CPE, 136 for NDM-CPE, and 92 for KPC-CPE. Patients colonized with OXA-CPE or NDM-CPE were younger (61.7±20 and 60.7±19.56, respectively) compared to those colonized with KPC-CPE (67.2±18.78; P=0.043 and P=0.013). Clinical characteristics and outcomes for the three cohorts are summarized in Table 1. Patients colonized with OXA-CPE or NDM-CPE were more likely to be admitted to surgical wards, have fewer urinary catheters and decubitus ulcers, and were more often discharged home compared to KPC-CPE colonized patients. OXA-CPE and NDM-CPE genes were predominately associated with Escherichia coli, while KPC-CPE gene was mainly found with Klebsiella sp. **Conclusions:** OXA-CPE and NDM-CPE colonized patients are younger, less debilitated and primarily reside at home. These findings prompted a revised CPE admission strategy, resulting in higher detection of OXA-CPE and NDM-CPE colonization upon admission.

**Figure:**
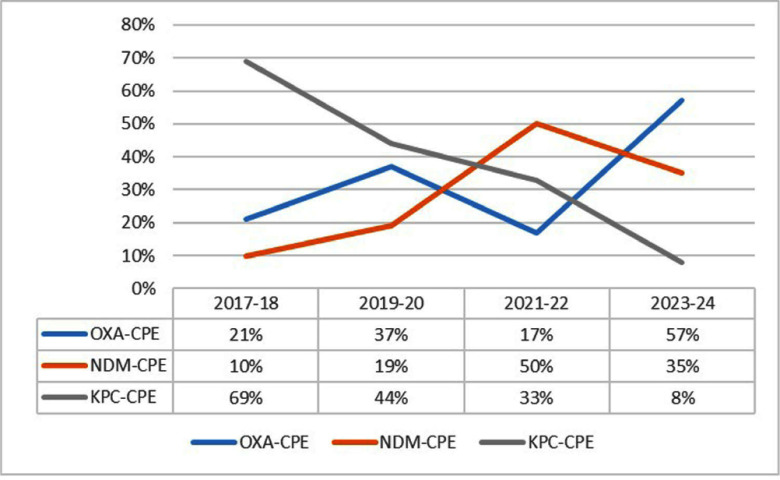

**Figure:**